# Neurovascular conflict patterns in glossopharyngeal neuralgia: a systematic review and meta-analysis of offending vessels and outcomes after microvascular decompression

**DOI:** 10.1007/s10143-026-04587-1

**Published:** 2026-11-14

**Authors:** Khushal Gupta, Maryam Zeinali, Joshua A. Benton, David Altschul, Vijay Agarwal, Michael Karsy

**Affiliations:** 1 The Leo M. Davidoff Department of Neurological Surgery, Montefiore Medical Center, Albert Einstein College of Medicine, 3316 Rochambeau Avenue, Bronx, NY 10467 USA; 2 Mazandaran University of Medical Sciences, Sari, Iran; 3 Department of Neurosurgery, University of Michigan, Ann Arbor, MI USA

**Keywords:** Glossopharyngeal neuralgia, Microvascular decompression, Neurovascular compression, Offending vessel, Posterior inferior cerebellar artery, Meta-analysis

## Abstract

Background: Microvascular decompression (MVD) for glossopharyngeal neuralgia (GPN) presumes a compressive neurovascular conflict at the glossopharyngeal-vagal root entry zone, but the distribution of offending vessels has never been quantified across series. Objective: To estimate the pooled prevalence of each offending vessel identified at MVD for GPN, alongside pain relief, recurrence, mortality and complications. Methods: PubMed/MEDLINE, Embase, Web of Science and Cochrane CENTRAL were searched from inception through August 2026. Studies with at least five GPN patients undergoing MVD and extractable outcomes were included. Proportions were pooled using the Freeman-Tukey double arcsine transformation and a DerSimonian-Laird random-effects model. Risk of bias was assessed with the Newcastle-Ottawa Scale (NOS). Results: Nineteen observational studies (1,000 MVD patients) were included, and methodological quality was acceptable to moderate (modified NOS median 5/6; comparative cohorts 6–7/9). Fifteen studies (674 patients) reported vessel-specific findings. The posterior inferior cerebellar artery (PICA) was implicated, alone or in combination, in 87% of patients (95% confidence interval [CI] 83–90), the least heterogeneous outcome (I² = 27.9%). Vertebral artery involvement was 12% (7–17), anterior inferior cerebellar artery involvement 7% (4–12) and venous conflict 3% (1–6). Multiple-vessel conflict was present in 14% (9–20). Immediate pain relief was 94% (92–97) and relief at last follow-up 90% (85–94) after a weighted mean follow-up of 66 months, with recurrence in 4% (1–9). Five deaths occurred (0.5%), four in earlier surgical eras. Sensitivity analyses for cohort overlap, rootlet section and unretrieved reports changed no estimate by 2 percentage points or more. Conclusion: The PICA was involved in about nine of ten patients with vessel data, the most consistent estimate in the review. About one in seven patients had more than one offending vessel, supporting full-length inspection of the IX-X rootlets rather than treatment of the first conflict encountered.

**Supplementary Information:**

The online version contains supplementary material available at https://doi.org/10.1007/s10143-026-04587-1.

## Introduction

Glossopharyngeal neuralgia (GPN), also termed vagoglossopharyngeal neuralgia, is a rare cranial neuralgia characterized by brief, severe, stabbing paroxysms in the throat, tonsillar fossa, base of tongue and deep ear, commonly triggered by swallowing, talking or coughing [1–4]. Secondary structural causes must be excluded [3, 5], and a subset of patients have bradycardia or syncope through the glossopharyngeal–vagal reflex arc [3, 6–8].

GPN typically relapses and remits. In the Mayo Clinic series of 217 patients, 161 reported spontaneous remissions [9], and in a population-based cohort from Rochester, Minnesota, the disease was generally mild and only a quarter of patients required surgery [10]. Long remissions, the rarity of the condition and misdiagnosis as trigeminal neuralgia or otological pain delay referral [11–13].

First-line treatment is carbamazepine or a related anticonvulsant, but many patients need a procedure because of inadequate pain control or intolerance [14–17]. Options include microvascular decompression (MVD), section of the glossopharyngeal and upper vagal rootlets (rhizotomy) and stereotactic radiosurgery [18–25]. Nerve-sparing MVD is generally preferred, with rootlet section reserved for complex or unclear conflicts [13, 19, 24, 26–29], and the principal morbidity is lower cranial nerve dysfunction [30–33].

Evidence for MVD comes from retrospective single-centre series with variable follow-up and outcome definitions [13, 24, 34, 35]. A recent meta-analysis pooled efficacy and safety and identified arterial compression as a favourable predictor of outcome [34], but the identity and number of offending vessels, which guide operative exposure and the judgement that decompression is complete, have not been quantified across series. We therefore performed a systematic review and single-arm meta-analysis of the offending vessels identified at MVD for GPN, together with pain relief, recurrence, mortality and complications.

## Methods

We conducted a systematic review and single-arm meta-analysis of MVD for GPN, designed a priori and reported in accordance with the PRISMA 2020 statement [36].

PubMed/MEDLINE, Embase, Web of Science and Cochrane CENTRAL were searched from inception to 23 August 2026 without language or publication-type restriction, using terms for the glossopharyngeal nerve and for neuralgia without intervention terms (Supplementary Table S1). Reference lists of eligible articles and reviews were hand-searched.

**Fig. 1:**
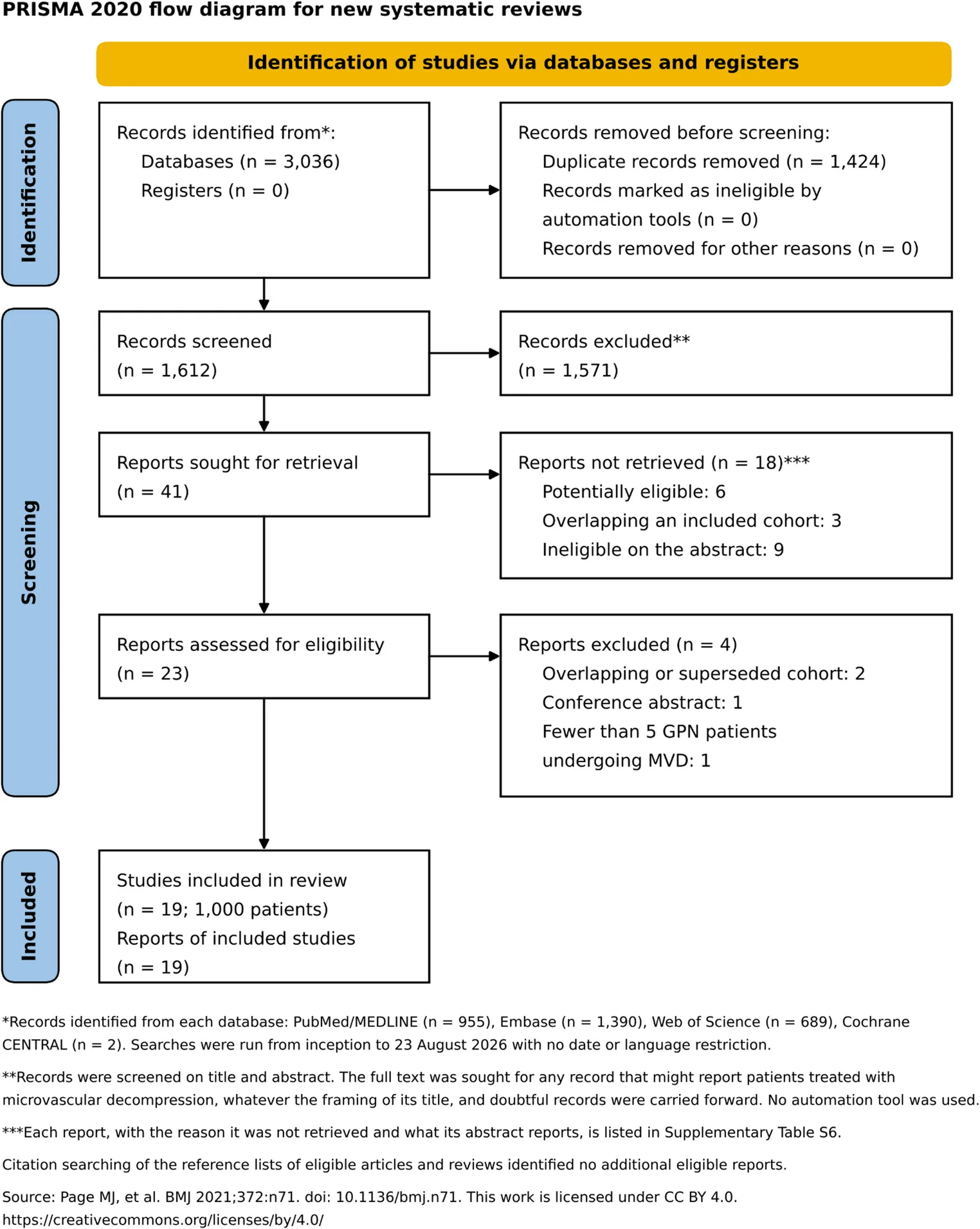
PRISMA 2020 flow diagram. GPN = glossopharyngeal neuralgia; MVD = microvascular decompression

Studies were eligible if they included ≥ 5 patients with GPN undergoing MVD, reported pain relief, recurrence, mortality or complications, and provided extractable data. From comparative studies, only the MVD-alone arm was used. Case reports, conference abstracts and non-surgical series were excluded. Reviewers independently screened titles, abstracts and full texts without automation tools, and disagreements were resolved by discussion. Full texts were sought through the institutional library, publishers’ websites and open-access sources, and the search was repeated on journal and publisher platforms for every potentially relevant report not retrieved. Reports excluded after full-text assessment and reports not retrieved are listed in Supplementary Tables S2 and S6 (Fig. 1).

Reports from the same centre were compared by recruitment period, and the report with the most complete vessel and outcome data, with priority to vessel data, was retained (Supplementary Table S3). Three Xinhua Hospital series were retained: Zhao et al. (January 2004 to June 2006) and Zheng X et al. (April 2013 to December 2018) do not overlap [37, 38], but the consecutive departmental series of Xia et al. (2006 to 2016, months not reported) could overlap them by up to 6 and 45 months, respectively [35], which was examined in sensitivity analyses.

The same reviewers extracted data independently, and the data were cross-checked against the source articles. Offending vessels were recorded as identified at surgery (or, in one series, on preoperative imaging [26]) and analysed as involvement alone or in combination, so the categories are not mutually exclusive. Outcomes followed each study’s definition, and long-term outcome was taken as the last reported follow-up. When an outcome was reported only for a mixed cohort, the MVD arm was isolated where possible. The one series that added rootlet section to MVD in every patient was retained and examined in a sensitivity analysis, and patients who underwent rootlet section at a reoperation were counted as failures of the index MVD. Medians with ranges or interquartile ranges were converted to estimated means [39], means were recomputed from patient-level tables where these were published, and in one series symptom duration was derived as mean age at surgery minus mean age at onset [40] (Supplementary Table S7). Investigators were not contacted.

The primary outcomes were immediate pain relief, pain relief at last follow-up, recurrence and mortality, and the secondary outcomes were the offending vessels and complications.

Risk of bias was assessed with the Newcastle–Ottawa Scale (NOS) [41], using a modified six-star version for single-arm series, in which the comparability domain does not apply, and the standard nine-star version for comparative cohorts (Supplementary Table S5). Each study was scored independently by the reviewers. Certainty of evidence was not graded, because uncontrolled single-arm series start at low certainty by design.

### Statistical analysis

Proportions were transformed with the Freeman–Tukey double arcsine [42], pooled with a DerSimonian–Laird random-effects model [43] to allow for differences in case mix, technique and follow-up between series, and back-transformed, with 95% confidence intervals (CI) and prediction intervals (PI). Heterogeneity was quantified with I² and τ². Subgroup analysis and meta-regression were not performed. Because only 3 of 19 studies recorded a death, mortality is reported as a crude rate. All analyses, including the sensitivity analyses, and the forest and funnel plots were produced in MetaAnalysisOnline.com [44].

Sensitivity analyses comprised leave-one-out analysis, exclusion of the comparative cohorts, exclusion of studies scoring 4/6 on the NOS, and exclusion of each Xinhua series in turn and of Zhao et al. and Zheng X et al. together. We also excluded the series with rootlet section at the index operation [45], alone and together with those using it at reoperation [29, 46, 47], replaced one retained report by a larger overlapping report from the same group (Supplementary Table S3) and added abstract-level data from potentially eligible reports not retrieved (Supplementary Tables S4 and S6). Funnel plots were inspected, recognizing that asymmetry can be artefactual when proportions approach 0 or 1.

## Results

### Study selection, characteristics and quality

Of 1,612 records screened, 41 reports were sought for full-text assessment. Eighteen could not be retrieved, and 4 of the 23 assessed were excluded (Fig. 1; Supplementary Tables S2 and S6), leaving 19 retrospective studies with 1,000 MVD patients [11–13, 26, 29, 30, 33, 35, 37, 38, 40, 45–52] (Table 1): 16 case series and three cohorts comparing MVD with rhizotomy or with MVD plus rhizotomy, published from 1998 to 2025 in eight countries. Sex was reported for 1,004 patients (59.7% women) rather than the 1,000 analysed, because four series reported it for a slightly different cohort [26, 46, 49, 51]. The weighted mean age was 57.3 years, symptom duration before surgery 5.1 years (17 studies; study means 3.1–8.5) and follow-up 65.9 months (16 studies; 15.7–155.8).

**Table 1:** Included study and patient characteristics

Study	Country / centre	Study design	*N*	Age (yrs)	Female	Symptom duration	Follow-up
Kondo 1998	Japan - Kitano Medical Research Institute, Osaka	Retrospective case series	20	59.3 (mean (40–75))	75.0% (12/16)	5.1 yrs	Mean 11.6 ± 2.8 yrs (5–16)
Patel 2002	USA - University of Pittsburgh	Retrospective case series	217	50.2 (mean ± 14.4)	66.8% (145/217)	5.7 yrs	Mean 48.8 mo (*n* = 50)
Sampson 2004	USA - Duke University (series of T. Fukushima, 1984–1991)	Retrospective case series	47	56.4 (mean (29–82))	61.7% (29/47)	7.1 yrs	Median 152 mo (124–211), *n* = 29
Ferroli 2009	Italy - Milan and Bologna	Retrospective case series	31	55.8 (mean† (27–88))	54.8% (17/31)	4.6 yrs	Mean 7.5 yrs (1–17)
Kandan 2010	UK - Frenchay Hospital, Bristol	Retrospective case series	10	52.5 (mean (34–81), whole cohort)	52.4% (11/21)	8.1 yrs (whole cohort)	Mean 4 yrs (1 mo-16 yrs), whole cohort
Kawashima 2010	Japan - Kyushu / Saga	Retrospective case series	14	59.2 (mean (42–73))	64.3% (9/14)	NR	Mean 6.5 yrs (1.1–11.8)
Gaul 2011	Germany - Erlangen-Nuremberg	Retrospective case series	18	54.5 (mean)	42.1% (8/19)	6.5 yrs	Some ≥ 10 yrs
Xiong 2012	China - Wuhan and Beijing	Retrospective case series	21	50.4 (mean (34–64))	61.9% (13/21)	8.5 yrs	Mean 3.4 yrs (1–5)
Kim 2017	South Korea - Ajou / Konkuk	Retrospective case series	30	52.4 (mean (31–72))	36.7% (11/30)	3.3 yrs	Mean 68.5 mo (25–251)
Zhao 2017	China - Xinhua, Shanghai	Retrospective case series	35	56.8 (mean ± 8.1†)	57.1% (20/35)	6.3 yrs†	Mean 132 mo (120–144), *n* = 30
Wang J 2018	China - Shandong University, Jinan	Retrospective comparative cohort	22	Range 40–81	54.5% (12/22)	NR	Median 48 mo (6 mo-6 yrs)
Xia 2018	China - Xinhua, Shanghai	Retrospective case series	228	63.2 (mean ± 11.7)	61.4% (140/228)	4.6 yrs	Mean 54.3 ± 6.2 mo
Zheng X 2020	China - Xinhua, Shanghai	Retrospective case series	46	61.6 (mean ± 11.8)	56.5% (26/46)	3.6 yrs	Mean 38 ± 17.9 mo (12–80)
Du 2021	China - Xuanwu, Beijing	Retrospective comparative cohort	67	55.3 (mean ± 10.3)	65.1% (41/63)	4.6 yrs	Mean 46.9 ± 20.9 mo (10–83)
Zheng W 2022	China - Peking Univ People’s, Beijing	Retrospective case series	97	59.8 (mean ± 10.7)	59.8% (58/97)	4.5 yrs	NR (pain-free survival 52 mo)
Wang L 2023	China - Tianjin Huanhu	Retrospective comparative cohort	39	62.9 (mean ± 9.2†)	46.2% (18/39)	3.1 yrs†	24–108 mo
Revuelta-Gutierrez 2023	Mexico - INNN, Mexico City	Retrospective case series	20	58.9 (mean ± 11.3)	60.0% (12/20)	7.6 yrs§	Mean 120.4 ± 67.3 mo (25–333)
Hurth 2024	Germany - Tuebingen and Essen	Retrospective case series	9	55.0 (mean ± 14.3)	66.7% (6/9)	4.8 yrs	Mean 15.7 ± 18.2 mo (2–53)
Joncas 2025	USA - Columbia University / UPMC	Retrospective case series	29	61.4 (estimated mean‡; median 65.0, IQR 52–68)	37.9% (11/29)	3.5 yrs‡	Mean 65.3 mo (0-134)
Total / pooled	19 studies, 8 countries	16 case series, 3 comparative cohorts	1,000	57.3 (weighted mean)	59.7% (599/1,004)	5.1 yrs (weighted mean)	65.9 mo (weighted mean)

N = patients in the MVD arm. Values are means unless stated. Sex was reported for a cohort that differed slightly from the MVD sample in four series (Kondo, Kandan, Gaul and Du), so the sex total is 1,004. †Recomputed from published patient-level data. ‡Estimated from the median and interquartile range [39]. §Mean age at surgery minus mean age at onset. Follow-up medians were converted to estimated means for the weighted mean. Derivations are given in Supplementary Table S7. *NR* not reported

**Fig. 2:**
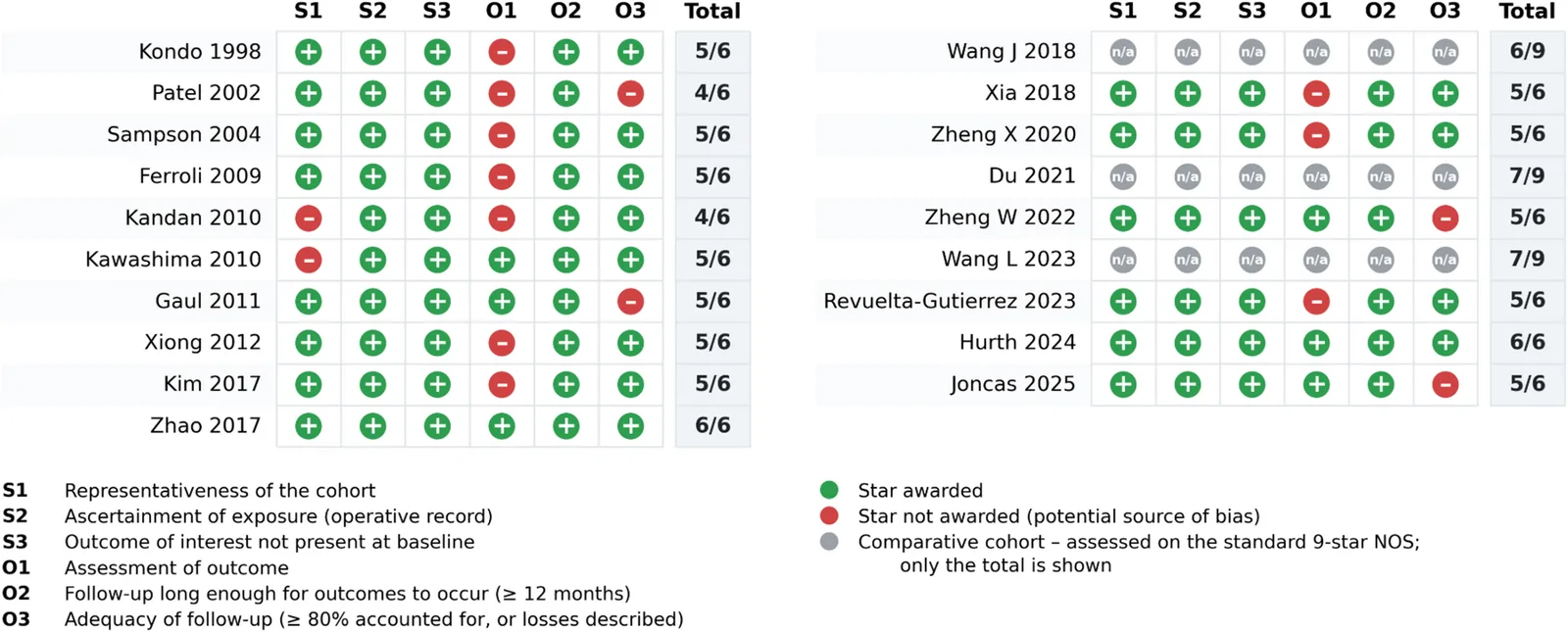
Methodological quality of the included studies (Newcastle–Ottawa Scale)

Single-arm series scored 4–6 of 6 on the modified NOS (median 5) and the comparative cohorts 6–7 of 9 (Fig. 2). Stars were lost mainly for outcome ascertainment by telephone and for incomplete follow-up.

### Offending vessels

**Fig. 3:**
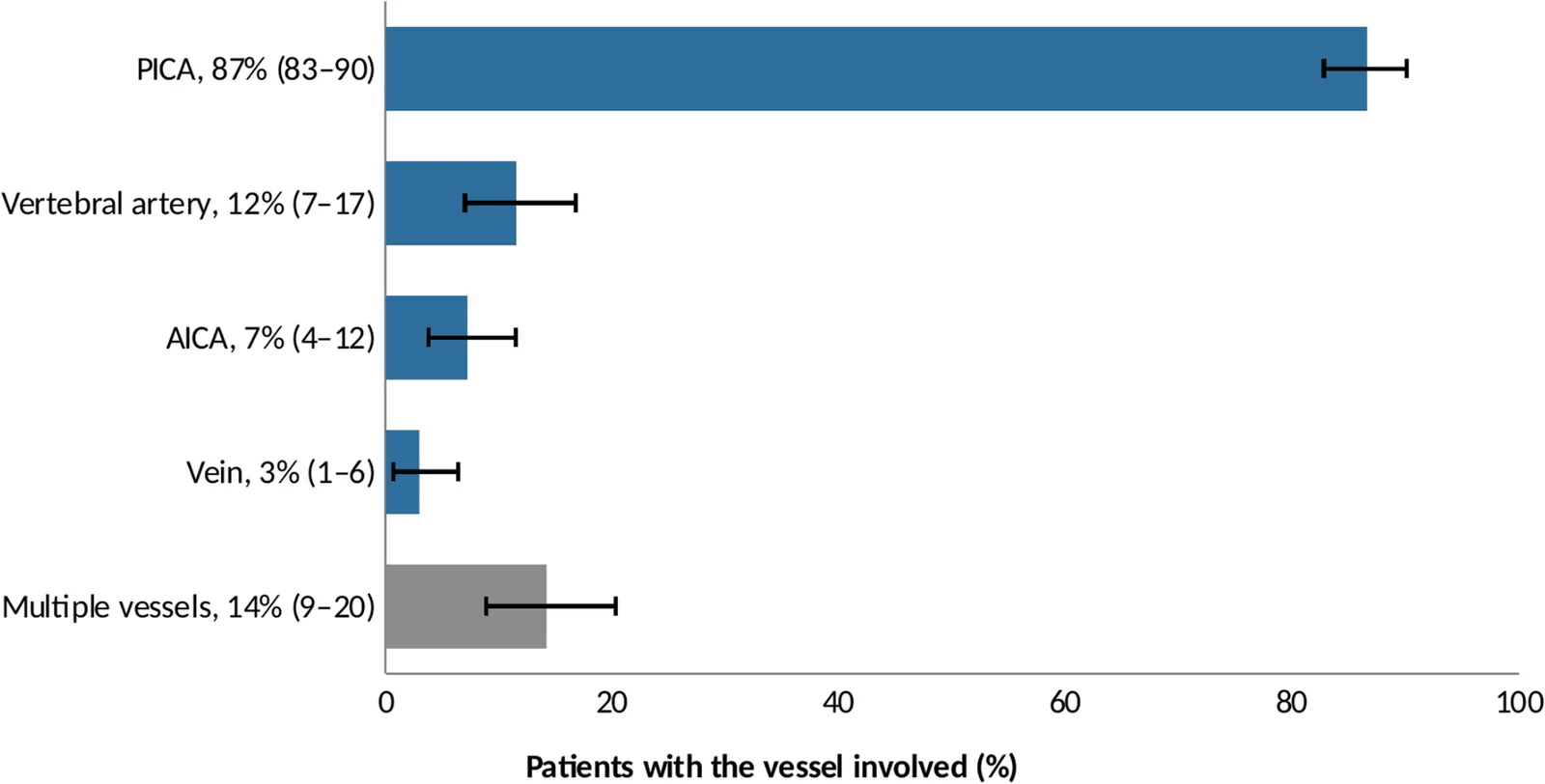
Offending vessels identified at microvascular decompression for glossopharyngeal neuralgia. Bars are pooled random-effects proportions from MetaAnalysisOnline.com with 95% CI (15 studies, 674 patients). Categories are not mutually exclusive. AICA = anterior inferior cerebellar artery; PICA = posterior inferior cerebellar artery

Fifteen studies (674 patients) reported vessels for the MVD arm (Table 2; Fig. 3, Supplementary Figure S2). The other four gave no counts or reported vessels only for combined MVD and rhizotomy cohorts [13, 46, 51, 52]. The posterior inferior cerebellar artery (PICA) was involved in 580 of 674 patients (pooled 87%, 95% CI 83–90%), the most consistent outcome (I² = 27.9%; PI 77–94%). Involvement of the vertebral artery, the anterior inferior cerebellar artery (AICA) and a vein was 12%, 7% and 3%, respectively, and 14% of patients had more than one offending vessel, usually the PICA with the vertebral artery or AICA.

**Table 2:** Distribution of offending vessels among studies reporting vessel-specific findings

Study	Patients with vessel data (*n*)	PICA, *n* (%)	VA, *n* (%)	AICA, *n* (%)	Venous, *n* (%)	Multiple vessels, *n* (%)	Other findings / comment
Kondo 1998	–	NR	NR	NR	NR	NR	Vessels described without counts
Patel 2002	–	NR	NR	NR	NR	NR	Vessels not reported
Sampson 2004	47	42 (89.4)	4 (8.5)	8 (17.0)	6 (12.8)	11 (23.4)	
Ferroli 2009	31	22 (71.0)	7 (22.6)	1 (3.2)	1 (3.2)	0 (0.0)	
Kandan 2010	10	8 (80.0)	4 (40.0)	1 (10.0)	0 (0.0)	3 (30.0)	
Kawashima 2010	14	12 (85.7)	0 (0.0)	4 (28.6)	0 (0.0)	2 (14.3)	
Gaul 2011	18	17 (94.4)	3 (16.7)	0 (0.0)	0 (0.0)	2 (11.1)	
Xiong 2012	21	18 (85.7)	4 (19.0)	6 (28.6)	0 (0.0)	7 (33.3)	
Kim 2017	30	27 (90.0)	3 (10.0)	2 (6.7)	2 (6.7)	8 (26.7)	Multiple also includes superior cerebellar artery (1) and small arteries (2)
Zhao 2017	35	28 (80.0)	5 (14.3)	0 (0.0)	1 (2.9)	0 (0.0)	Choroid plexus (1)
Wang J 2018	–	NR	NR	NR	NR	NR	Whole cohort only (MVD + rhizotomy)
Xia 2018	228	204 (89.5)	24 (10.5)	14 (6.1)	27 (11.8)	39 (17.1)	
Zheng X 2020	46	39 (84.8)	9 (19.6)	6 (13.0)	1 (2.2)	11 (23.9)	Small arteries only (2)
Du 2021	–	NR	NR	NR	NR	NR	Whole cohort only (MVD + rhizotomy)
Zheng W 2022	97	79 (81.4)	4 (4.1)	9 (9.3)	15 (15.5)	10 (10.3)	
Wang L 2023	39	32 (82.1)	9 (23.1)	2 (5.1)	0 (0.0)	4 (10.3)	
Revuelta-Gutierrez 2023	20	15 (75.0)	1 (5.0)	0 (0.0)	0 (0.0)	3 (15.0)	PICA with superior cerebellar artery (2); choroid plexus (2); vertebrobasilar complex (2); none found (1)
Hurth 2024	9	9 (100.0)	3 (33.3)	0 (0.0)	0 (0.0)	3 (33.3)	
Joncas 2025	29	28 (96.6)	0 (0.0)	5 (17.2)	0 (0.0)	4 (13.8)	
Pooled, % (95% CI)	674	87 (83–90)	12 (7–17)	7 (4–12)	3 (1–6)	14 (9–20)	I² = 27.9%, 63.7%, 59.1%, 61.6%, 69.0%

Vessels are counted as involvement alone or in combination, so categories are not mutually exclusive. Pooled estimates are random-effects proportions. *NR* not reported. *AICA* anterior inferior cerebellar artery, *PICA* posterior inferior cerebellar artery, *VA* vertebral artery

### Pain relief, recurrence and mortality

**Fig. 4:**
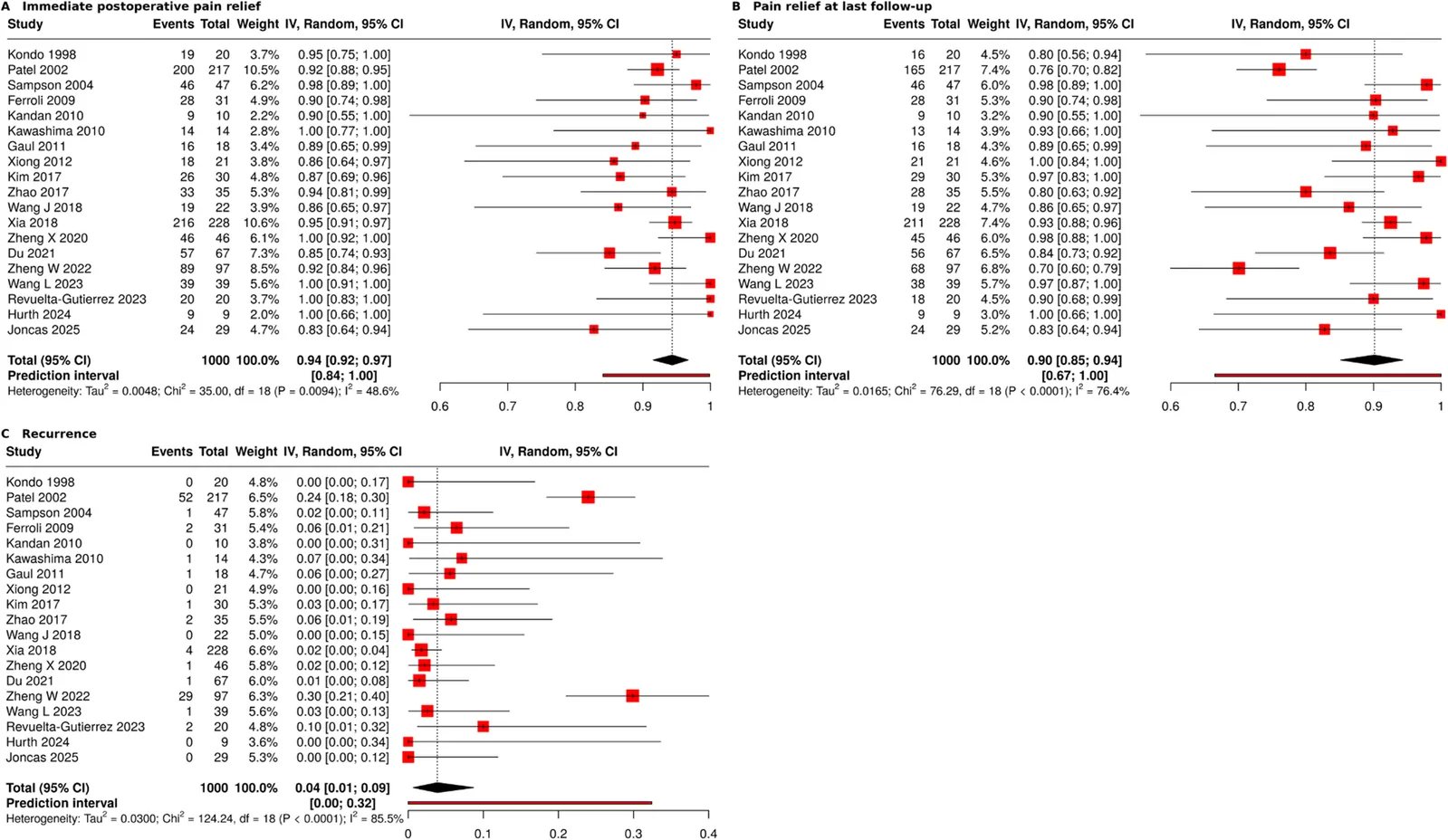
Pain relief and recurrence after microvascular decompression for glossopharyngeal neuralgia. (**A**) Immediate pain relief; (**B**) relief at last follow-up; (**C**) recurrence (19 studies, 1,000 patients). Squares are study proportions sized by weight with 95% CI. Diamonds are pooled random-effects estimates, and red bars show 95% prediction intervals

Immediate pain relief was 94% (95% CI 92–97%), relief at last follow-up 90% (85–94%) and recurrence 4% (1–9%) (Fig. 4; Table 3). Five deaths occurred in three studies [13, 29, 51], a crude mortality of 0.5% (Supplementary Figure S1).

**Table 3:** Pain outcomes, recurrence, mortality and complications

Study	Immediate pain relief	Relief at last follow-up	Recurrence	Mortality	Complications
Kondo 1998	19/20 (95.0%)	16/20 (80.0%)	0/20 (0.0%)	1/20 (5.0%)	6 transient CN IX-X palsy; 2 persistent hoarseness; 1 death (superior sagittal sinus thrombosis)
Patel 2002	200/217 (92.2%)	165/217 (76.0%)	52/217 (24.0%)	3/217 (1.4%)	CN IX-X palsy 9.6% falling to 2.9%; operative death and intracranial haematoma 5.8% in 1973–1987, none thereafter; CSF leak < 2% in the modern era
Sampson 2004	46/47 (97.9%)	46/47 (97.9%)	1/47 (2.1%)	0/47 (0.0%)	34% immediate deficits; 11% permanent (hoarseness, dysphagia, facial paresis)
Ferroli 2009	28/31 (90.3%)	28/31 (90.3%)	2/31 (6.5%)	0/31 (0.0%)	3 transient dysphagia/dysphonia; 4 transient hypoacusia; 1 CSF rhinorrhoea
Kandan 2010	9/10 (90.0%)	9/10 (90.0%)	0/10 (0.0%)	0/10 (0.0%)	2 persistent hoarseness; 1 CSF leak; no mortality
Kawashima 2010	14/14 (100.0%)	13/14 (92.9%)	1/14 (7.1%)	0/14 (0.0%)	4 transient hoarseness/dysphagia; 2 slight persistent; no mortality
Gaul 2011	16/18 (88.9%)	16/18 (88.9%)	1/18 (5.6%)	0/18 (0.0%)	2 CSF hypersecretion; 1 DVT/PE; 6 transient CN IX-X deficits
Xiong 2012	18/21 (85.7%)	21/21 (100.0%)	0/21 (0.0%)	0/21 (0.0%)	Posterior tongue and pharyngeal hypoaesthesia in all (rhizotomy); transient dizziness (3), tinnitus (1), facial weakness (1) and hearing loss (1); no mortality
Kim 2017	26/30 (86.7%)	29/30 (96.7%)	1/30 (3.3%)	0/30 (0.0%)	7/30 transient (23.3%); 2 CSF rhinorrhoea; 1 hearing loss; no mortality
Zhao 2017	33/35 (94.3%)	28/35 (80.0%)	2/35 (5.7%)	0/35 (0.0%)	1 CSF leak; 1 delayed facial palsy (recovered); no long-term morbidity; no surgical mortality
Wang J 2018	19/22 (86.4%)	19/22 (86.4%)	0/22 (0.0%)	0/22 (0.0%)	2 transient hoarseness/cough; 1 CSF leak; 1 intracranial infection; 1 hearing loss; no mortality
Xia 2018	216/228 (94.7%)	211/228 (92.5%)	4/228 (1.8%)	0/228 (0.0%)	37 transient deficits (16.2%); 2 permanent (0.88%)
Zheng X 2020	46/46 (100.0%)	45/46 (97.8%)	1/46 (2.2%)	0/46 (0.0%)	1 wound healing problem; 1 hearing loss
Du 2021	57/67 (85.1%)	56/67 (83.6%)	1/67 (1.5%)	0/67 (0.0%)	CN complications 3.0% (vs. 50.0% after rhizotomy); no mortality
Zheng W 2022	89/97 (91.8%)	68/97 (70.1%)	29/97 (29.9%)	0/97 (0.0%)	25 (25.8%) immediate complications, including 2 CSF leaks; 9 (9.3%) permanent (dysphagia, hoarseness, facial paresis, hearing loss); no mortality
Wang L 2023	39/39 (100.0%)	38/39 (97.4%)	1/39 (2.6%)	1/39 (2.6%)	2 postoperative haemorrhage (1 death); 1 swallowing and coughing difficulty; 1 CSF rhinorrhoea
Revuelta-Gutierrez 2023	20/20 (100.0%)	18/20 (90.0%)	2/20 (10.0%)	0/20 (0.0%)	CSF leak and meningitis reported; no mortality
Hurth 2024	9/9 (100.0%)	9/9 (100.0%)	0/9 (0.0%)	0/9 (0.0%)	No permanent deficits; no mortality
Joncas 2025	24/29 (82.8%)	24/29 (82.8%)	0/29 (0.0%)	0/29 (0.0%)	10% immediate and 11% long-term complications; no mortality
Pooled (19 studies)	94% (92–97)	90% (85–94)	4% (1–9)	5/1,000 (0.5%), crude	Transient CN IX–X deficits most frequent, 0–34% across studies

Outcomes follow each study’s definitions (in some series, recurrence denotes any long-term failure), so the outcome columns need not reconcile. Pooled proportions are random-effects estimates, and mortality is the crude rate. *CN* cranial nerve, *CSF* cerebrospinal fluid

### Complications

Transient lower cranial nerve deficits were the commonest complication (0–34%). In the larger series that reported them, permanent deficits, mostly hoarseness or dysphagia, affected 0.9% to 11% of patients [33, 35, 47], and cerebrospinal fluid leaks were reported in nine series (Table 3). Four of the five deaths occurred in earlier surgical eras: three during 1973–1987 in the Pittsburgh series, whose operative mortality and intracranial haematoma rates of 5.8% then fell to zero [13], and one from sinus thrombosis early in a series begun in 1980 [51]. The fifth followed postoperative haemorrhage in a contemporary series [29].

### Rootlet section

Only one series added rootlet section to MVD in every patient, with immediate relief in 18 of 21 and relief at last follow-up in 21 of 21 [45]. In the comparative cohorts, rhizotomy alone or with MVD gave relief similar to MVD alone but more lower cranial nerve morbidity, including permanent hoarseness in 2 of 15 patients [52], long-term complications in 50.0% versus 3.0% [46] and IX–X dysfunction in 8 of 22 versus 1 of 39 [29]. Rootlet section at reoperation after a failed MVD was reported in nine patients from three series [29, 46, 47].

### Sensitivity analyses

No sensitivity analysis moved any estimate by 2 percentage points or more (Supplementary Table S4). Leave-one-out ranges were 93.7–95.0% for immediate relief, 89.4–91.3% for relief at last follow-up and 2.9–4.2% for recurrence. Excluding a Xinhua series changed multiple-vessel conflict most (14.2% to 15.8% without Zhao et al.), excluding the series with rootlet section changed no estimate by more than 1.1 percentage points, and adding six potentially eligible reports that were not retrieved (76 patients) gave immediate relief of 95.5% and relief at last follow-up of 91.7%. PICA involvement remained the least heterogeneous outcome in every sensitivity analysis.

### Prognostic factors

Recurrence or reoperation was associated with incomplete decompression [35, 47, 48], absence of a clear conflict [33] and mixed or atypical pain [13]. In 97 patients, venous compression (multivariable hazard ratio [HR] 8.2) and low-grade conflict (HR 5.5) predicted shorter pain-free survival, whereas a symptom duration of 2–6 years (univariable HR 0.77, 95% CI 0.32–1.88) or of more than 6 years (HR 0.71, 0.24–2.12) did not, compared with less than 2 years [47]. No other study analysed outcome by symptom duration.

## Discussion

MVD gave immediate relief in 94% of patients and lasting relief in 90% at a weighted mean follow-up of more than five years, with 4% recurrence and 0.5% mortality. These estimates agree closely with the recent meta-analysis by Hajikarimloo et al. (25 studies, 1,009 patients; initial and long-term complete relief 94.2% and 92.1%) [34]. Because the two study sets only partly overlap, this agreement suggests that the efficacy of MVD does not depend on which series are pooled. Our higher recurrence estimate (4% versus 2.3%) most plausibly reflects differences in follow-up and in how recurrence was defined.

The two reviews answer different questions. The earlier synthesis modelled conflict as arterial or venous [34] and did not establish which vessels are responsible or how often more than one vessel is involved, the information a surgeon needs to plan exposure and judge the completeness of decompression. We also restricted pooling to MVD arms, documented overlapping cohorts and tested the influence of overlap, rootlet section and unretrieved reports. The PICA was involved in 87% of patients with the lowest heterogeneity of any outcome, despite differences in era, region and reporting, consistent with the high-origin PICA loop that compresses the glossopharyngeal nerve from below [50]. Exposure should therefore anticipate a PICA conflict at the IX–X root entry zone, while vertebral artery involvement (12%) and multiple offenders (14%) argue for inspecting the full length of the rootlets rather than treating the first vessel encountered, an approach emphasized by the Xinhua group, whose series reported low recurrence rates [35, 38]. Venous conflict was uncommon but was associated with failure in a multivariable analysis [47], and the only unrelieved patient in another series had a vein encircling the nerve [33].

The natural history of GPN affects both referral and the interpretation of outcomes. Spontaneous remissions occur in about three quarters of patients [9] and the disease is often mild [10]. These features, together with initial control by anticonvulsants and misdiagnosis [11–13], help explain the mean interval of 5.1 years before surgery. Remissions also complicate observational follow-up, because a pain-free interval after MVD may coincide with a remission and a late recurrence may reflect the disease rather than a failed decompression, so short follow-up can overstate durable success. In trigeminal neuralgia, symptoms lasting more than 8 years predicted recurrence after MVD [53], and chronic compression causes focal demyelination at the root entry zone [54], a plausible substrate for less reversible neuropathic pain. In GPN, the only study to examine duration found no association [47], but it was not designed for this question. Prospective registries recording symptom duration, remission history and time to surgery are needed to test whether earlier MVD prevents a transition to fixed neuropathic pain.

Because rootlet section was confined to one series at the index operation and to reoperations elsewhere, and excluding these series changed no estimate by more than 1.1 percentage points, the pooled results reflect decompression rather than ablation. The comparative data support rhizotomy for a negative exploration or when safe decompression is not feasible, rather than as a routine adjunct [24, 29].

Lower cranial nerve deficits dominate morbidity [24]. Catastrophic events clustered in earlier surgical eras, when operative mortality and haematoma in the Pittsburgh series were 5.8% before falling to zero [13]. A contemporary death after haemorrhage [29] shows that the risk persists, particularly when dissection near perforators or the brainstem is needed [28, 48].

This review has limitations. All studies were retrospective, with heterogeneous outcome definitions and follow-up, partly by telephone [13, 35, 48]. Vessel data were missing for 326 of 1,000 patients, including the Pittsburgh series [13], and vessels were attributed by unblinded surgeons or, in one series, on imaging [26]. Up to an estimated 37 patients may be counted twice through the Xinhua series, and 18 reports, half of them not in English, were not retrieved, although neither changed the estimates materially. Mortality was too rare to pool, and funnel-plot asymmetry (Supplementary Figure S3) is expected for proportions near 0 or 1. Prospective multicentre registries with fixed follow-up, standard pain scales and explicit reporting of offending vessels would address these gaps.

## Conclusion

MVD gives near-universal immediate relief of GPN and durable relief in about nine of ten patients. The PICA is involved in 87% of patients, and one patient in seven has more than one offending vessel, supporting inspection of the full length of the IX–X rootlets. Morbidity is mainly transient lower cranial nerve dysfunction, and perioperative death has become rare.

## Supplementary Information

Below is the link to the electronic supplementary material.

Supplementary Material 1

Supplementary Material 2

## Data Availability

All data extracted from the included studies are presented in the article and its Supplementary Material: the full search string for each database in Supplementary Table S1, the reports excluded at full-text assessment with their reasons in Supplementary Table S2, the overlapping cohorts identified and the report retained for each centre in Supplementary Table S3, the pooled estimates, heterogeneity statistics and sensitivity analyses for every outcome in Supplementary Table S4, the item-level risk-of-bias scores in Supplementary Table S5, the reports that were not retrieved in Supplementary Table S6 and the derivation of study-level age, symptom duration and follow-up in Supplementary Table S7. The complete data-extraction spreadsheet and the data as entered for each analysis in MetaAnalysisOnline.com are available from the corresponding author on reasonable request. The PRISMA 2020 checklist accompanies this submission.

## Declarations

### Use of artificial intelligence

AI-assisted tools were used in the preparation of this manuscript. All content was reviewed and accepted by the authors, who take full responsibility for the work.

### Prior presentation

Preliminary results of this review were presented at the 2026 Annual Meeting of the North American Skull Base Society and published in abstract form (Gupta K, Karsy M. Glossopharyngeal neuralgia treatment with microvascular decompression: a single-arm meta-analysis of pain relief durability and signals. J Neurol Surg B Skull Base 2026;87. https://doi.org/10.1055/s-0046-1819384). The present manuscript is a substantial expansion of that abstract: the search was re-run and broadened, the included study set enlarged, and the offending-vessel synthesis, the Newcastle-Ottawa assessment and the sensitivity analyses appear here for the first time. Where pooled estimates differ from those in the abstract, the values reported here supersede them.

### Registration and protocol

This review was not registered and a formal protocol was not prepared. The eligibility criteria, outcomes and primary analysis plan were defined a priori and are reported in full in the Methods. The sensitivity analyses addressing cohort overlap, rootlet section and unretrieved reports were added during peer review.

### Clinical trial number

Clinical trial number: not applicable.

## References

[1] Blumenfeld A, Nikolskaya G (2013) Glossopharyngeal neuralgia. Curr Pain Headache Rep 17:343. 10.1007/s11916-013-0343-x 23709235

[2] Headache Classification Committee of the International Headache Society (IHS) (2018) The international classification of headache disorders, 3rd edition. Cephalalgia 38:1–211. 10.1177/0333102417738202 29368949

[3] Pearce JM (2006) Glossopharyngeal neuralgia. Eur Neurol 55:49–52. 10.1159/000091429 16479122

[4] Singh PM, Kaur M, Trikha A (2013) An uncommonly common: Glossopharyngeal neuralgia. Ann Indian Acad Neurol 16:1–8. 10.4103/0972-2327.107662 PMC3644765 23661955

[5] Boeddinghaus R, Whyte A (2021) Imaging of trigeminal neuralgia and other facial pain. Neuroimaging Clin N Am 31:485–508. 10.1016/j.nic.2021.05.008 34689929

[6] Krasoudakis A, Anyfantakis D, Hadjipetrou A, Kastanakis M, Symvoulakis EK, Marathianos S (2015) Glossopharyngeal neuralgia associated with cardiac syncope: Two case reports and literature review. Int J Surg Case Rep 12:4–6. 10.1016/j.ijscr.2015.05.007 PMC4485693 25974354

[7] Ozenci M, Karaoguz R, Conkbayir C, Altin T, Kanpolat Y (2003) Glossopharyngeal neuralgia with cardiac syncope treated by glossopharyngeal rhizotomy and microvascular decompression. Europace 5:149–152. 10.1053/eupc.2002.0298 12633639

[8] Savica R, Lagana A, Calabro RS, Casella C, Musolino R (2007) Vagoglossopharyngeal neuralgia: a rare case of sincope responding to pregabalin. Cephalalgia 27:566–567. 10.1111/j.1468-2982.2007.01304.x 17598795

[9] Rushton JG, Stevens JC, Miller RH (1981) Glossopharyngeal (vagoglossopharyngeal) neuralgia: a study of 217 cases. Arch Neurol 38:201–205. 10.1001/archneur.1981.00510040027002 7213143

[10] Katusic S, Williams DB, Beard CM, Bergstralh E, Kurland LT (1991) Incidence and clinical features of glossopharyngeal neuralgia, Rochester, Minnesota, 1945–1984. Neuroepidemiology 10:266–275. 10.1159/000110283 1798429

[11] Hurth H, Roder C, Tatagiba M, Ebner FH (2024) Diagnostic and treatment pitfalls in glossopharyngeal neuralgia: evidence from a case series. Acta Neurochir (Wien) 166:415. 10.1007/s00701-024-06313-6 39422793

[12] Kim MK, Park JS, Ahn YH (2017) Microvascular decompression for glossopharyngeal neuralgia: Clinical analyses of 30 cases. J Korean Neurosurg Soc 60:738–748. 10.3340/jkns.2017.0506.010 PMC5678068 29142635

[13] Patel A, Kassam A, Horowitz M, Chang Y-F (2002) Microvascular decompression in the management of glossopharyngeal neuralgia: analysis of 217 cases. Neurosurgery 50:705–711. 10.1097/00006123-200204000-00004 11904019

[14] Han A, Montgomery C, Zamora A, Winder E, Kaye A, Carroll C et al (2022) Glossopharyngeal neuralgia: Epidemiology, risk factors, pathophysiology, differential diagnosis, and treatment options. Health Psychol Res 10:36042. 10.52965/001c.36042 PMC9239365 35774913

[15] Park JS, Ahn YH (2023) Glossopharyngeal Neuralgia. J Korean Neurosurg Soc 66:12–23. 10.3340/jkns.2022.0178 PMC9837485 36480819

[16] Rohde MA, Viera LGA, Meneses AO, Rypl HF, Nunes LP, Annes VHW et al (2026) Treatment of glossopharyngeal neuralgia: a systematic review. Pain Pract 26:e70144. 10.1111/papr.70144 41793136

[17] Rozen TD (2004) Trigeminal neuralgia and glossopharyngeal neuralgia. Neurol Clin 22:185–206. 10.1016/s0733-8619(03)00094-x 15062534

[18] Bauman MMJ, Bayan SL, Peris-Celda M (2025) Staged bilateral microsurgical rhizotomy for treatment of bilateral glossopharyngeal neuralgia: illustrative case. J Neurosurg Case Lessons 10:CASE25359. 10.3171/case25359 PMC12477898 41021989

[19] Berckemeyer MA, Suarez-Meade P, Carcelen MFV, Ricci MD, Cheshire WP, Trifiletti DM et al (2023) Current advances in the surgical treatment of glossopharyngeal neuralgia. Neurosurg Rev 46:47. 10.1007/s10143-023-01948-y 36725770

[20] Franzini A, Messina G, Franzini A, Marchetti M, Ferroli P, Fariselli L et al (2017) Treatments of glossopharyngeal neuralgia: towards standard procedures. Neurol Sci 38:51–55. 10.1007/s10072-017-2909-6 28527060

[21] Hajikarimloo B, Mohammadzadeh I, Mortezaei A, Ebrahimi A, Habibi MA (2025) Radiosurgery for Glossopharyngeal Neuralgia: A Systematic Review and Meta-Analysis. World Neurosurg 202:124389. 10.1016/j.wneu.2025.124389 40816446

[22] Lévêque M, Park MC, Melhaoui A, Yomo S, Donnet A, Régis J (2011) Gamma knife radiosurgery for glossopharyngeal neuralgia: Marseille experience. J Radiosurg SBRT 1:41–46 PMC5658899 29296296

[23] Pommier B, Touzet G, Lucas C, Vermandel M, Blond S, Reyns N (2018) Glossopharyngeal neuralgia treated by Gamma Knife radiosurgery: safety and efficacy through long-term follow-up. J Neurosurg 128:1372–1379. 10.3171/2017.3.jns162542 28621622

[24] Rey-Dios R, Cohen-Gadol AA (2013) Current neurosurgical management of glossopharyngeal neuralgia and technical nuances for microvascular decompression surgery. Neurosurg Focus 34:E8. 10.3171/2012.12.focus12391 23451790

[25] Siempis T, Rehder R, Voulgaris S, Alexiou GA (2024) Stereotactic radiosurgery for idiopathic glossopharyngeal neuralgia: A systematic review. World Neurosurg X 22:100325. 10.1016/j.wnsx.2024.100325 PMC10918277 38455249

[26] Gaul C, Hastreiter P, Duncker A, Naraghi R (2011) Diagnosis and neurosurgical treatment of glossopharyngeal neuralgia: clinical findings and 3-D visualization of neurovascular compression in 19 consecutive patients. J Headache Pain 12:527–534. 10.1007/s10194-011-0349-x PMC3173624 21567138

[27] Laha RK, Jannetta PJ (1977) Glossopharyngeal neuralgia. J Neurosurg 47:316–320. 10.3171/jns.1977.47.3.0316 894337

[28] Resnick DK, Jannetta PJ, Bissonnette D, Jho HD, Lanzino G (1995) Microvascular decompression for glossopharyngeal neuralgia. Neurosurgery 36:64–68. 10.1227/00006123-199501000-00008 7708170

[29] Wang L, Liu Q, Dong X, Wang J (2023) Comparative analysis of MVD and RHZ in the treatment of primary glossopharyngeal neuralgia: A clinical report on 61 cases. Front Neurol 14:1024142. 10.3389/fneur.2023.1024142 PMC9968861 36860578

[30] Joncas CT, Ma K, Tugend M, Washington E, Di Stefano G, Truini A et al (2025) Long-term outcomes after microvascular decompression for glossopharyngeal neuralgia. Neurosurg Focus 59:E19. 10.3171/2025.6.focus25287 PMC12987579 40889403

[31] Lu VM, Goyal A, Graffeo CS, Perry A, Jonker BP, Link MJ (2018) Glossopharyngeal neuralgia treatment outcomes after nerve section, microvascular decompression, or stereotactic radiosurgery: A systematic review and meta-analysis. World Neurosurg 120:572–582.e7. 10.1016/j.wneu.2018.09.042 30240868

[32] Revuelta-Gutiérrez R, Morales-Martínez AH, Mejías-Soto C, Martínez-Anda JJ, Ortega-Porcayo LA (2016) Microvascular decompression for glossopharyngeal neuralgia through a microasterional approach: A case series. Surg Neurol Int 7:51. 10.4103/2152-7806.181824 PMC4866068 27213105

[33] Sampson JH, Grossi PM, Asaoka K, Fukushima T (2004) Microvascular decompression for glossopharyngeal neuralgia: long-term effectiveness and complication avoidance. Neurosurgery 54:884–889. 10.1227/01.neu.0000114142.98655.cc 15046654

[34] Hajikarimloo B, Mohammadzadeh I, Tos SM, Mortezaei A, Habibi MA (2026) Microvascular decompression in glossopharyngeal neuralgia: a systematic review and meta-analysis. Acta Neurol Belg 126:793–807. 10.1007/s13760-025-02978-7 41428184

[35] Xia L, Li YS, Liu MX, Zhong J, Dou NN, Li B et al (2018) Microvascular decompression for glossopharyngeal neuralgia: a retrospective analysis of 228 cases. Acta Neurochir (Wien) 160:117–123. 10.1007/s00701-017-3347-1 29103137

[36] Page MJ, McKenzie JE, Bossuyt PM, Boutron I, Hoffmann TC, Mulrow CD et al (2021) The PRISMA 2020 statement: an updated guideline for reporting systematic reviews. BMJ 372:n71. 10.1136/bmj.n71 PMC8005924 33782057

[37] Zhao H, Zhang X, Zhu J, Tang YD, Li ST (2017) Microvascular decompression for glossopharyngeal neuralgia: Long-term follow-up. World Neurosurg 102:151–156. 10.1016/j.wneu.2017.02.106 28263933

[38] Zheng X, Wei X-Y, Zhu J, Yuan Y, Ying T-T, Li S-T (2020) Microvascular decompression alone without rhizotomy is an effective way of treating glossopharyngeal neuralgia: Clinical analysis of 46 cases. Stereotact Funct Neurosurg 98:129–135. 10.1159/000505712 32101860

[39] Luo D, Wan X, Liu J, Tong T (2018) Optimally estimating the sample mean from the sample size, median, mid-range, and/or mid-quartile range. Stat Methods Med Res 27:1785–1805. 10.1177/0962280216669183 27683581

[40] Revuelta-Gutierrez R, Pinon-Jimenez F, Contreras-Vazquez OR, Vales-Hidalgo LO, Martinez-Anda JJ (2023) Glossopharyngeal and vagoglossopharyngeal neuralgia: long-term surgical outcomes in a single institution. Surg Neurol Int 14:267. 10.25259/sni_518_2023 PMC10408601 37560568

[41] Stang A (2010) Critical evaluation of the Newcastle-Ottawa scale for the assessment of the quality of nonrandomized studies in meta-analyses. Eur J Epidemiol 25:603–605. 10.1007/s10654-010-9491-z 20652370

[42] Freeman MF, Tukey JW (1950) Transformations related to the angular and the square root. Ann Math Stat 21:607–611. 10.1214/aoms/1177729756

[43] DerSimonian R, Laird N (1986) Meta-analysis in clinical trials. Control Clin Trials 7:177–188. 10.1016/0197-2456(86)90046-2 3802833

[44] Fekete JT, Győrffy B (2025) MetaAnalysisOnline.com: Web-based tool for the rapid meta-analysis of clinical and epidemiological studies. J Med Internet Res 27:e64016. 10.2196/64016 PMC11926438 39928123

[45] Xiong NX, Zhao HY, Zhang FC, Liu RE (2012) Vagoglossopharyngeal neuralgia treated by microvascular decompression and glossopharyngeal rhizotomy: clinical results of 21 cases. Stereotact Funct Neurosurg 90:45–50. 10.1159/000333828 22249175

[46] Du T, Ni B, Shu W, Hu Y, Zhu H, Li Y (2021) Neurosurgical choice for glossopharyngeal neuralgia: A benefit-harm assessment of long-term quality of life. Neurosurgery 88:131–139. 10.1093/neuros/nyaa325 32735682

[47] Zheng W, Zhao P, Song H, Liu B, Zhou J, Fan C et al (2022) Prognostic factors for long-term outcomes of microvascular decompression in the treatment of glossopharyngeal neuralgia: a retrospective analysis of 97 patients. J Neurosurg 137:820–827. 10.3171/2021.9.jns21877 34920419

[48] Ferroli P, Fioravanti A, Schiariti M, Tringali G, Franzini A, Calbucci F et al (2009) Microvascular decompression for glossopharyngeal neuralgia: a long-term retrospectic review of the Milan-Bologna experience in 31 consecutive cases. Acta Neurochir (Wien) 151:1245–1250. 10.1007/s00701-009-0330-5 19513582

[49] Kandan SR, Khan S, Jeyaretna DS, Lhatoo S, Patel NK, Coakham HB (2010) Neuralgia of the glossopharyngeal and vagal nerves: long-term outcome following surgical treatment and literature review. Br J Neurosurg 24:441–446. 10.3109/02688697.2010.487131 20726751

[50] Kawashima M, Matsushima T, Inoue T, Mineta T, Masuoka J, Hirakawa N (2010) Microvascular decompression for glossopharyngeal neuralgia through the transcondylar fossa (supracondylar transjugular tubercle) approach. Neurosurgery 66:275–280. 10.1227/01.neu.0000369662.36524.cf 20489516

[51] Kondo A (1998) Follow-up results of using microvascular decompression for treatment of glossopharyngeal neuralgia. J Neurosurg 88:221–225. 10.3171/jns.1998.88.2.0221 9452227

[52] Wang J, Yu R, Qu C, Jiang J, Wang C, Meng Q et al (2018) Does glossopharyngeal neuralgia need rhizotomy in neurovascular decompression surgery? J Craniofac Surg 29:2192–2194. 10.1097/scs.0000000000004856 30320682

[53] Barker FG 2nd, Jannetta PJ, Bissonette DJ, Larkins MV, Jho HD (1996) The long-term outcome of microvascular decompression for trigeminal neuralgia. N Engl J Med 334:1077–1083. 10.1056/NEJM199604253341701 8598865

[54] Love S, Coakham HB (2001) Trigeminal neuralgia: pathology and pathogenesis. Brain 124:2347–2360. 10.1093/brain/124.12.2347 11701590

