# Supplementary material for: Neurovascular conflict patterns in glossopharyngeal neuralgia: a systematic review and meta-analysis of offending vessels and outcomes after microvascular decompression
Source: Neurosurg Rev. 2026 Nov 14;49(1):651. doi: 10.1007/s10143-026-04587-1 (PMC13768215; doi:10.1007/s10143-026-04587-1)
Supplement: Supplementary file 1 [file 10143_2026_4587_MOESM1_ESM.docx]

**Supplementary Material**

*Neurovascular Conflict Patterns in Glossopharyngeal Neuralgia: A Systematic Review and Meta-Analysis of Offending Vessels and Outcomes After Microvascular Decompression*

**Contents**

Supplementary Table S1. Full electronic search strategy

Supplementary Table S2. Reports excluded at full-text assessment

Supplementary Table S3. Reports from the same centres, recruitment periods and the report retained

Supplementary Table S4. Pooled estimates and sensitivity analyses

Supplementary Table S5. Newcastle–Ottawa risk-of-bias assessment

Supplementary Table S6. Reports sought but not retrieved

Supplementary Table S7. Derivation of study-level age, symptom duration and follow-up

Supplementary Figure S1. Perioperative mortality: forest and funnel plots

Supplementary Figure S2. Forest plots of offending-vessel involvement

Supplementary Figure S3. Funnel plots for the eight pooled outcomes

**Supplementary Table S1. Full electronic search strategy.**

| **Database** | **Search string** | **Records** |
| --- | --- | --- |
| PubMed/MEDLINE (pubmed.ncbi.nlm.nih.gov); searched 23 Aug 2026 | ("Glossopharyngeal Nerve Diseases"[Mesh] OR glossopharyngeal*[tiab] OR glosso-pharyngeal*[tiab] OR vagoglossopharyngeal*[tiab] OR vago-glossopharyngeal*[tiab] OR glossopharyngeus[tiab] OR "ninth cranial nerve"[tiab] OR "9th cranial nerve"[tiab] OR "cranial nerve IX"[tiab]) AND ("Neuralgia"[Mesh] OR "Facial Pain"[Mesh] OR "Trigeminal Neuralgia"[Mesh] OR neuralgi*[tiab] OR neuralgic[tiab] OR "facial pain"[tiab] OR "craniofacial pain"[tiab] OR "orofacial pain"[tiab] OR "throat pain"[tiab] OR otalgia[tiab] OR "tic douloureux"[tiab]) | 955 |
| Embase (embase.com); searched 23 Aug 2026 | ('glossopharyngeal neuralgia'/exp OR glossopharyngeal*:ti,ab,kw OR 'glosso-pharyngeal':ti,ab,kw OR vagoglossopharyngeal*:ti,ab,kw OR glossopharyngeus:ti,ab,kw OR 'ninth cranial nerve':ti,ab,kw OR 'cranial nerve IX':ti,ab,kw) AND ('neuralgia'/exp OR 'face pain'/exp OR neuralgi*:ti,ab,kw OR 'facial pain':ti,ab,kw OR 'craniofacial pain':ti,ab,kw OR 'orofacial pain':ti,ab,kw OR 'throat pain':ti,ab,kw OR otalgia:ti,ab,kw OR 'tic douloureux':ti,ab,kw) | 1,390 |
| Web of Science (Core Collection); searched 23 Aug 2026 | TS=(glossopharyngeal* OR "glosso-pharyngeal*" OR vagoglossopharyngeal* OR "vago-glossopharyngeal*" OR glossopharyngeus OR "ninth cranial nerve" OR "9th cranial nerve" OR "cranial nerve IX") AND TS=(neuralgi* OR "facial pain" OR "craniofacial pain" OR "orofacial pain" OR "throat pain" OR otalgia OR "tic douloureux") | 689 |
| Cochrane CENTRAL (cochranelibrary.com); searched 23 Aug 2026 | (glossopharyngeal* OR vagoglossopharyngeal*) AND (neuralgi* OR "facial pain") [Title Abstract Keyword] | 2 |

*Searches were run on 23 August 2026 from database inception with no date, language or publication-type restriction. Total records identified = 3,036; 1,424 duplicates removed; 1,612 records screened.*

**Supplementary Table S2. Reports excluded at full-text assessment (n = 4).**

| **Report** | **Reason for exclusion** |
| --- | --- |
| Taha JM, Tew JM Jr (1995) Long-term results of surgical treatment of idiopathic neuralgias of the glossopharyngeal and vagal nerves. Neurosurgery 36:926-931 | Only 4 patients underwent microvascular decompression (threshold ≥5), all with concurrent rhizotomy; no offending vessels reported. |
| Segura-Zenón AF, Munguía-Rodríguez AG, Torres-Torres YR, et al (2022) Microvascular decompression for glossopharyngeal neuralgia: a single-center experience. J Neurol Surg B Skull Base 83(S01). https://doi.org/10.1055/s-0042-1743843 | Conference abstract; not a full report. |
| Revuelta-Gutiérrez R, Morales-Martínez AH, Mejías-Soto C, et al (2016) Microvascular decompression for glossopharyngeal neuralgia through a microasterional approach: a case series. Surg Neurol Int 7:51 | Superseded by the same institution’s fuller report (Revuelta-Gutierrez 2023, n = 20, 1994-2023), which includes this cohort. |
| Wakiya K, Fukushima T, Miyazaki S (1989) Results of microvascular decompression in 16 cases of glossopharyngeal neuralgia. Neurol Med Chir (Tokyo) 29:1113-1118. https://doi.org/10.2176/nmc.29.1113 | Overlapping cohort: 16 patients treated over a 4-year period at Mitsui Memorial Hospital, Tokyo, by the surgeon (T. Fukushima) whose consecutive 1984-1991 series of 47 patients is reported in full by Sampson et al. 2004, the report retained. |

**Supplementary Table S3. Reports from the same centres, with recruitment periods and the report retained.**

| **Centre** | **Report** | **Recruitment period and patients** | **Relationship and decision** |
| --- | --- | --- | --- |
| University of Pittsburgh, USA | Patel 2002 | 1973-2000; 217 MVD | Retained (fullest report of the series). |
|  | Laha 1977; Resnick 1995 | 6 patients (1970s); 40 MVD since 1971 | Earlier reports of the same series, largely contained in Patel 2002; excluded. |
|  | Habeych 2014; Thirumala 2015 | January 2008-August 2012, 27 MVD; 6 GPN patients within a mixed MVD audiometry cohort (period not reported) | Later reports; no pain-relief or vessel outcomes; excluded (Habeych 2014 is also listed in Supplementary Table S6). |
| Columbia University and UPMC, USA (R.F. Sekula) | Joncas 2025 | 2013-2021; 29 consecutive MVD by the senior author | Retained. |
|  | Jani 2018 | MRI evaluation protocol from the same surgeon’s practice (period and number not reported) | No extractable outcomes; excluded. |
| T. Fukushima (Sampson 2004 reported from Duke University, USA; Wakiya 1989 from Mitsui Memorial Hospital, Tokyo, Japan) | Sampson 2004 | 1984-1991; 47 consecutive MVD | Retained. |
|  | Wakiya 1989 | 16 MVD over 4 years, published 1989 | Same surgeon and period; excluded (Supplementary Table S2). |
| Xinhua Hospital, Shanghai Jiao Tong University, China | Zhao 2017 | January 2004-June 2006; 35 MVD | Retained. |
|  | Xia 2018 | 2006-2016 (months not reported); 228 MVD, consecutive departmental series | Retained. |
|  | Zheng X 2020 | April 2013-December 2018; 46 MVD | Retained. |
|  | Wang YN 2014 | 2007-2013; 6 patients with coexisting trigeminal neuralgia, hemifacial spasm and GPN | Within the Xia 2018 period; excluded. |
| Kyushu University and Saga University, Japan (T. Matsushima) | Kawashima 2010 | 1997-2008; 14 MVD | Retained: offending vessel, outcome and follow-up reported for every operated patient. |
|  | Inoue 2025 | 2003-2019; 23 MVD, with vessels and outcomes reported for the 21 followed for more than 5 years | Overlaps Kawashima 2010 for 2003-2008; excluded. Substitution for Kawashima 2010 in all analyses is shown in Supplementary Table S4. |
|  | Matsushima 2010; Inoue 2020 | Technical report of the same approach; anatomical study of 18 patients operated over 10 years (vessels only) | No pain-relief, recurrence, mortality or complication data; excluded. |
| University of Erlangen-Nuremberg, Germany | Gaul 2011 | 1994-2009; 18 MVD | Retained. |
|  | Tanrikulu 2015 | 15 GPN patients, prospective imaging study (period not reported) | Same department and imaging programme; excluded. |
| Xuanwu Hospital, Capital Medical University, Beijing, China | Du 2021 | November 2012-December 2018; 67 MVD patients (63 MVD alone, 4 rhizotomy after failed MVD) | Retained. |
|  | Ni 2020 | 8 reoperations after failed MVD, same department (period not reported in the abstract) | Overlap with Du 2021; not retrieved (Supplementary Table S6). |
|  | Liang 2017 | July 2011-October 2016; 18 MVD, Department of Neurosurgery | Overlap with Du 2021 cannot be excluded; not retrieved (Supplementary Table S6). |
|  | Gao 2007 | 12 patients, published 2007; imaging study | No overlap; not retrieved (Supplementary Table S6). |
| National Institute of Neurology and Neurosurgery, Mexico City | Revuelta-Gutierrez 2023 | 1994-2023; 20 MVD | Retained. |
|  | Revuelta-Gutiérrez 2016 | 14 consecutive MVD over the preceding 20 years | Superseded; excluded (Supplementary Table S2). |
| Tianjin Huanhu Hospital, China | Wang L 2023 | March 2013-March 2020; 39 MVD, 22 rhizotomy | Retained (MVD arm). |
|  | Wang L 2024 (Chinese Journal of Neurosurgery) | Period stated as March 2014-March 2021; 61 patients (39 MVD, 22 rhizotomy) with the same haemorrhages, death and recurrence | Duplicate report of the same 61 patients, although the period is stated one year later; not retrieved (Supplementary Table S6). |
| Peking University People’s Hospital, Beijing, China | Zheng W 2022 (J Neurosurg) | January 2010-July 2019; 97 MVD | Retained. |
|  | Zheng W 2022 (Chinese Journal of Neurosurgery) | January 2014-November 2019; 61 MVD | Largely overlaps the J Neurosurg report (January 2014-July 2019 lies within its period); not retrieved (Supplementary Table S6). |
| Ajou University, Suwon, South Korea | Kim 2017 | March 1996-December 2016; 30 MVD with at least 2 years of follow-up | Retained. |
|  | Ahn 2024 | Conference abstract; 82 patients, 1996-2023 | Overlap with Kim 2017 and conference abstract; not retrieved (Supplementary Table S6). |

*Recruitment periods and patient numbers are given where reported. Reports listed here but not in Supplementary Table S2 or S6 were excluded at title and abstract screening, because the abstract identified them as earlier, later or partial reports of an included cohort or showed that they reported no extractable outcomes. Zhao 2017 and Zheng X 2020 do not overlap: Zheng X 2020 compared its 46 new patients with the earlier Zhao cohort. Xia 2018 could overlap Zhao 2017 by up to 6 months (January-June 2006) and Zheng X 2020 by up to 45 months (April 2013-December 2016); assuming uniform recruitment, at most about 7 and 30 patients could be counted twice. The effect of excluding each of these series is shown in Supplementary Table S4. Kawashima 2010 and the larger later report of Inoue 2025 (Inoue K, Matsushima T, Maeyama H, Goto Y, Ohara S, Abe T. Neurosurg Focus 2025;59:E20; https://doi.org/10.3171/2025.6.FOCUS25424) overlap only for 2003-2008. Kawashima 2010 was retained because it reports the offending vessel, outcome and follow-up for every operated patient, whereas Inoue 2025 reports pooled data for the 21 of 23 patients followed for more than 5 years: PICA alone in 14, AICA alone in 5, PICA with the vertebral artery in 1 and PICA with AICA in 1; immediate relief in 20, one recurrence treated by reoperation and complete relief at last follow-up in the other 20.*

**Supplementary Table S4. Pooled estimates and sensitivity analyses.**

**(a) Pooled estimates for all outcomes**

| **Outcome** | **Studies** | **Patients** | **Pooled % (95% CI)** | **95% PI** | **I²** | **τ²** |
| --- | --- | --- | --- | --- | --- | --- |
| Immediate pain relief | 19 | 1,000 | 94.3 (91.5-96.7) | 84.1-99.9 | 48.6% | 0.0048 |
| Pain relief at last follow-up | 19 | 1,000 | 90.1 (85.1-94.3) | 66.5-100.0 | 76.4% | 0.0165 |
| Recurrence | 19 | 1,000 | 3.9 (0.7-8.7) | 0.0-32.4 | 85.5% | 0.0300 |
| Mortality | 19 | 1,000 | 0.5 (crude rate, 5/1,000) | - | - | - |
| PICA involvement | 15 | 674 | 86.6 (82.8-90.1) | 77.1-94.2 | 27.9% | 0.0024 |
| Vertebral artery involvement | 15 | 674 | 11.5 (7.0-16.8) | 0.3-31.5 | 63.7% | 0.0107 |
| AICA involvement | 15 | 674 | 7.2 (3.8-11.5) | 0.0-23.4 | 59.1% | 0.0088 |
| Venous involvement | 15 | 674 | 3.0 (0.7-6.4) | 0.0-17.2 | 61.6% | 0.0098 |
| Multiple-vessel conflict | 15 | 674 | 14.2 (8.9-20.3) | 0.5-38.0 | 69.0% | 0.0136 |

**(b) Sensitivity analyses: pooled % for each outcome**

| **Analysis** | **k (efficacy / vessels)** | **Immediate relief** | **Relief at last follow-up** | **Recurrence** | **PICA** | **VA** | **AICA** | **Venous** | **Multiple** |
| --- | --- | --- | --- | --- | --- | --- | --- | --- | --- |
| Primary analysis | 19 / 15 | 94.3 | 90.1 | 3.9 | 86.6 | 11.5 | 7.2 | 3.0 | 14.2 |
| Leave-one-out analysis (range) | 18 / 14 | 93.7-95.0 | 89.4-91.3 | 2.9-4.2 | 85.6-87.7 | 10.6-12.7 | 6.4-8.2 | 2.2-3.6 | 13.2-15.8 |
| Excluding the three comparative cohorts | 16 / 14 | 94.7 | 90.2 | 4.6 | 87.0 | 10.6 | 7.5 | 3.6 | 14.6 |
| Excluding the two studies scoring 4/6 on the modified NOS | 17 / 14 | 94.5 | 91.0 | 3.2 | 86.7 | 10.7 | 7.2 | 3.3 | 13.7 |
| Excluding Zheng X 2020 | 18 / 14 | 93.7 | 89.5 | 4.0 | 86.7 | 10.9 | 6.8 | 3.1 | 13.5 |
| Excluding Zhao 2017 | 18 / 14 | 94.3 | 90.7 | 3.8 | 87.1 | 11.4 | 8.2 | 3.0 | 15.8 |
| Excluding Zhao 2017 and Zheng X 2020 | 17 / 13 | 93.7 | 90.0 | 3.9 | 87.2 | 10.7 | 7.8 | 3.1 | 15.2 |
| Excluding Xia 2018 | 18 / 14 | 94.2 | 89.9 | 4.1 | 85.6 | 11.9 | 7.5 | 2.3 | 14.1 |
| Excluding Xiong 2012 (rootlet section at the index operation) | 18 / 14 | 94.6 | 89.4 | 4.2 | 86.6 | 11.1 | 6.4 | 3.4 | 13.2 |
| Excluding Xiong 2012 and the three series with rootlet section at reoperation (Du 2021, Zheng W 2022, Wang L 2023) | 15 / 12 | 95.0 | 90.4 | 3.3 | 87.7 | 11.2 | 6.2 | 3.1 | 14.0 |
| Replacing Kawashima 2010 by the partly overlapping later report of the same group (Inoue 2025) | 19 / 15 | 94.1 | 90.3 | 3.8 | 86.2 | 11.8 | 7.5 | 3.0 | 13.9 |
| Adding abstract-level data from six potentially eligible reports not retrieved | 22-25 / 16-17 | 95.5 | 91.7 | 3.4 | 87.1 | 11.2 | 7.5 | 3.1 | 14.3 |

*All analyses were run in MetaAnalysisOnline.com (random-effects model, inverse-variance method, Freeman–Tukey double arcsine transformation, DerSimonian–Laird estimator); estimates are as reported by the platform to three decimal places. Vessel categories are counted as involvement alone or in combination and are not mutually exclusive. Mortality is reported as a crude rate because only 3 of 19 studies recorded a death and the back-transformation is degenerate at this event rate. The final analysis adds the abstract-level data of the six potentially eligible reports that were not retrieved (Supplementary Table S6): Sindou 1991 (8 MVD-alone patients; immediate and last follow-up relief), Zhao K 2000 (5 MVD-alone patients; relief and recurrence), Gao 2007 (12; immediate relief), Liang 2017 (18; immediate and last follow-up relief only, because the series was selected for PICA conflict), Qian 2026 (24; relief, recurrence and vertebral artery, venous and multiple-vessel involvement) and Grigoryan 2026 (9; all outcomes). The substitution analysis replaces Kawashima 2010 by the data of Inoue 2025 (21 patients followed for more than 5 years; Supplementary Table S3) for all outcomes. k = number of studies, given as the range across the efficacy and vessel outcomes. AICA = anterior inferior cerebellar artery; PICA = posterior inferior cerebellar artery; VA = vertebral artery.*

**Supplementary Table S5. Newcastle–Ottawa risk-of-bias assessment for all 19 included studies.**

| **Study** | **NOS version** | **S1** | **S2** | **S3** | **O1** | **O2** | **O3** | **Total** | **Comment** |
| --- | --- | --- | --- | --- | --- | --- | --- | --- | --- |
| Kondo 1998 | Modified (6) | ★ | ★ | ★ | – | ★ | ★ | 5/6 | Long-term outcome by postal questionnaire. |
| Patel 2002 | Modified (6) | ★ | ★ | ★ | – | ★ | – | 4/6 | Long-term outcome by telephone survey in 50 of 217 patients (23%). |
| Sampson 2004 | Modified (6) | ★ | ★ | ★ | – | ★ | ★ | 5/6 | Long-term outcome by direct contact with 29 of 47 patients (62%), or with the family or physician if the patient had died. |
| Ferroli 2009 | Modified (6) | ★ | ★ | ★ | – | ★ | ★ | 5/6 | Long-term outcome partly by telephone survey. |
| Kandan 2010 | Modified (6) | – | ★ | ★ | – | ★ | ★ | 4/6 | MVD-only subgroup of a mixed glossopharyngeal/vagal neuralgia cohort; long-term outcome by telephone. |
| Kawashima 2010 | Modified (6) | – | ★ | ★ | ★ | ★ | ★ | 5/6 | Patients selected for a specific transcondylar fossa technique. |
| Gaul 2011 | Modified (6) | ★ | ★ | ★ | ★ | ★ | – | 5/6 | Follow-up duration not reported systematically for the whole cohort. |
| Xiong 2012 | Modified (6) | ★ | ★ | ★ | – | ★ | ★ | 5/6 | Follow-up by telephone and/or clinic visit. |
| Kim 2017 | Modified (6) | ★ | ★ | ★ | – | ★ | ★ | 5/6 | Long-term outcome by telephone. |
| Zhao 2017 | Modified (6) | ★ | ★ | ★ | ★ | ★ | ★ | 6/6 |  |
| Wang J 2018 | Standard (9) | 3/4 |  |  | 1/2 | 2/3 |  | 6/9 | Comparative series; groups not adjusted for baseline pain duration. |
| Xia 2018 | Modified (6) | ★ | ★ | ★ | – | ★ | ★ | 5/6 | Long-term outcome by telephone survey. |
| Zheng X 2020 | Modified (6) | ★ | ★ | ★ | – | ★ | ★ | 5/6 | Follow-up by telephone interview or outpatient visit. |
| Du 2021 | Standard (9) | 3/4 |  |  | 2/2 | 2/3 |  | 7/9 |  |
| Zheng W 2022 | Modified (6) | ★ | ★ | ★ | ★ | ★ | – | 5/6 | Outcome assessed by blinded independent observers; follow-up duration not stated (pain-free survival reported instead). |
| Wang L 2023 | Standard (9) | 3/4 |  |  | 2/2 | 2/3 |  | 7/9 |  |
| Revuelta-Gutierrez 2023 | Modified (6) | ★ | ★ | ★ | – | ★ | ★ | 5/6 | Follow-up from the last clinical note and/or by telephone. |
| Hurth 2024 | Modified (6) | ★ | ★ | ★ | ★ | ★ | ★ | 6/6 |  |
| Joncas 2025 | Modified (6) | ★ | ★ | ★ | ★ | ★ | – | 5/6 | Follow-up range includes patients with 0 months of follow-up. |

*Single-arm series were scored on a modified six-star NOS covering the selection and outcome/follow-up domains; the comparability domain does not apply in the absence of a control group. S1 representativeness of the cohort; S2 ascertainment of exposure from the operative record; S3 outcome of interest not present at baseline; O1 assessment of outcome (star when the outcome was taken from clinical records or clinic review or was assessed by blinded independent observers; no star when it relied wholly or partly on patient or proxy report obtained by telephone, post or direct contact); O2 follow-up long enough for outcomes to occur (mean or median follow-up ≥ 12 months); O3 adequacy of follow-up (≥ 80% of the cohort accounted for, or losses described). ★ = star awarded, – = star not awarded. The three comparative cohorts were scored on the standard nine-star NOS and their domain subtotals are given as selection/comparability/outcome in the S1, O1 and O2 columns respectively.*

**Supplementary Table S6. Reports sought but not retrieved (n = 18).**

| **Report** | **Language** | **Design and population (from the abstract)** | **Reason the full text was not assessed** | **Assessment** |
| --- | --- | --- | --- | --- |
| POTENTIALLY ELIGIBLE, INDEPENDENT OR UNCERTAIN COHORT (n = 6) | | | | |
| Sindou M, Henry JF, Blanchard P (1991) Idiopathic neuralgia of the glossopharyngeal nerve: study of a series of 14 cases and review of the literature. Neurochirurgie. PMID 2017288 | French | 14 patients (Lyon): MVD in 9 (8 alone, 1 with rootlet section), rootlet section alone in 2, thermocoagulation in 3; total relief after every direct procedure; mean follow-up 3.5 years; vessels not counted in the abstract | No electronic full text located (French-language print journal) | Potentially eligible (8 MVD-alone patients); abstract data used in sensitivity analysis |
| Zhao K, Zuo H, Zhang L, Yu Y, Yuan Y, Guo J (2000) Long-term follow-up results of microsurgical treatment for glossopharyngeal neuralgia. Zhonghua Wai Ke Za Zhi. PMID 11832117 | Chinese | 21 patients, 1986-1998: MVD 5, MVD with rhizotomy 4, rhizotomy 12; all pain-free after surgery; no recurrence at a mean of 7.2 years | Chinese-language full text not available through institutional access or open sources | Potentially eligible (5 MVD-alone patients); abstract data used in sensitivity analysis |
| Gao YA, Shi WW, Liu HL, Wang ZW, Yin JX, Li KC, et al (2007) Glossopharyngeal neuralgia: MR findings. Chinese Journal of Radiology | Chinese | Imaging study of 12 patients treated with MVD (8 with concurrent trigeminal neuralgia); neuralgia resolved in all | As above | Potentially eligible (imaging focus); abstract data (immediate relief) used in sensitivity analysis |
| Liang J, Li M, Chen G, Guo H, Li Z, Bao Y (2017) Microvascular decompression for glossopharyngeal neuralgia with posterior inferior cerebellar artery as offending artery. Chinese Journal of Cerebrovascular Diseases 14:94-97. https://doi.org/10.3969/j.issn.1672-5921.2017.02.007 | Chinese | 18 consecutive MVD patients (Xuanwu Hospital, July 2011-October 2016), all with PICA conflict; 17 pain-free, 1 unrelieved; follow-up 1-62 months | As above | Potentially eligible; overlap with Du 2021 (same hospital) cannot be excluded; abstract data used in sensitivity analysis |
| Qian Z, Dai X, Zhao S, Ma C, Chai S, Xiong N (2026) Efficacy of individualized microvascular decompression guided by offending vessel classification for glossopharyngeal neuralgia. Chinese Journal of Neurosurgery. https://doi.org/10.3760/cma.j.cn112050-20250920-00367 | Chinese (English abstract) | 24 MVD patients (Zhongnan Hospital, Wuhan, September 2020-January 2025): small artery 20, composite with vertebral artery 2, vein 2; immediate relief 24/24; 23/24 at a mean of 24.2 months | Full text on the Chinese Medical Association platform not accessible through institutional access | Potentially eligible; abstract data used in sensitivity analysis |
| Grigoryan GY, Usachev DY, Grigoryan YA (2026) Vascular decompression in the treatment of vagoglossopharyngeal neuralgia. Zh Vopr Neirokhir Im N N Burdenko 90:15-20. https://doi.org/10.17116/neiro20269004115 | Russian (English abstract) | 9 patients: PICA 6, PICA with AICA 2, PICA with vertebral artery 1; complete relief in all; no recurrence at a mean of 8.5 years | Full text available only by individual purchase from the publisher | Potentially eligible; abstract data used in sensitivity analysis |
| OVERLAPPING AN INCLUDED COHORT (n = 3) | | | | |
| Wang L, Li P, Dong X, Wang J, Liu Q (2024) Analysis of clinical characteristics and therapeutic efficacy of 61 cases of primary drug-refractory glossopharyngeal neuralgia. Chinese Journal of Neurosurgery. https://doi.org/10.3760/cma.j.cn112050-20230416-00114 | Chinese | 61 patients (Tianjin Huanhu Hospital, period stated as March 2014-March 2021): MVD 39, rhizotomy 22; the same haemorrhages, death and recurrence as Wang L 2023 | Chinese-language full text not available through institutional access or open sources | Duplicate report of the 61 patients in Wang L 2023 (included) |
| Zheng W, Liu J, Hao Q, Gao X, Wu G, Fang J, et al (2022) Clinical efficacy and influencing factor analysis of microvascular decompression for primary glossopharyngeal neuralgia. Chinese Journal of Neurosurgery. https://doi.org/10.3760/cma.j.cn112050-20220120-00044 | Chinese | 61 MVD patients (Peking University People’s Hospital, January 2014-November 2019) | As above | Largely overlaps Zheng W 2022, J Neurosurg (included; January 2010-July 2019, n = 97) |
| Ni B, Hu YS, Du T, Zhang XH, Zhu HW (2020) Reoperation after failed microvascular decompression for glossopharyngeal neuralgia. Acta Neurochir (Wien). https://doi.org/10.1007/s00701-020-04383-w | English | 8 reoperations after failed MVD (Xuanwu Hospital) | Full text not obtained; on re-examination the abstract establishes ineligibility | Reoperation series from the department of Du 2021 (included); not a primary MVD cohort |
| INELIGIBLE ON THE ABSTRACT (n = 9) | | | | |
| Ahn YH, Yoo HJ, Cho SA (2024) Surgical treatment of glossopharyngeal neuralgia; surgical outcome of microvascular decompression using transposition techniques to achieve “off the root entry zone”. Stereotact Funct Neurosurg (meeting abstract) | English | Conference abstract: 83 MVD procedures in 82 patients, 1996-2023; 96.4% pain-free | Conference abstract; no full report identified | Ineligible (conference abstract); overlaps Kim 2017 (same senior author and institution) |
| Shankar V, Shreya VS, Vyas H, et al (2020) Frameless radiosurgery for intractable idiopathic glossopharyngeal neuralgia. Int J Radiat Oncol Biol Phys (meeting abstract). https://doi.org/10.1016/j.ijrobp.2020.07.1506 | English | Linear accelerator radiosurgery in 7 patients | Meeting-abstract supplement not obtained | Ineligible (radiosurgery; conference abstract) |
| Kano H, Urgosik D, Liscak R, et al (2016) Stereotactic radiosurgery for idiopathic glossopharyngeal neuralgia: an international multicenter study. J Neurosurg 125(Suppl 1):147-153. https://doi.org/10.3171/2016.7.GKS161523 | English | Gamma Knife radiosurgery in 22 patients | Full text not obtained; on re-examination the abstract establishes ineligibility | Ineligible (no MVD arm) |
| Martínez-Alvarez R, Martínez-Moreno N, Kusak ME, Rey-Portolés G (2014) Glossopharyngeal neuralgia and radiosurgery. J Neurosurg. https://doi.org/10.3171/2014.8.GKS141273 | English | Gamma Knife radiosurgery in 5 patients | Full text not obtained; on re-examination the abstract establishes ineligibility | Ineligible (no MVD arm) |
| Garcia Callejo FJ, Marco Algarra J, Talamantes Escriba F, et al (1999) Use of gabapentin in glossopharyngeal neuralgia. Acta Otorrinolaringol Esp. PMID 10217696 | Spanish | Gabapentin in 7 patients | Full text not obtained; on re-examination the abstract establishes ineligibility | Ineligible (pharmacological treatment) |
| Shin JH, Herrera SR, Eboli P, et al (2009) Entrapment of the glossopharyngeal nerve in patients with Eagle syndrome: surgical technique and outcomes in a series of 5 patients. J Neurosurg. https://doi.org/10.3171/2009.1.JNS08485 | English | Styloidectomy in 5 patients with Eagle syndrome | Full text not obtained; on re-examination the abstract establishes ineligibility | Ineligible (no MVD; Eagle syndrome) |
| Honey CM, Krüger MT, Rheaume AR, et al (2020) Concurrent glossopharyngeal neuralgia and hemi-laryngopharyngeal spasm (HeLPS): a case report and a review of the literature. Neurosurgery. https://doi.org/10.1093/neuros/nyz546 | English | Single case and literature review | Full text not obtained; on re-examination the abstract establishes ineligibility | Ineligible (case report) |
| Ferrante L, Artico M, Nardacci B, et al (1995) Glossopharyngeal neuralgia with cardiac syncope. Neurosurgery 36:58-63. https://doi.org/10.1227/00006123-199501000-00007 | English | 5 patients with syncope (of 15 with GPN) treated by rootlet section or MVD | Full text not obtained; on re-examination the abstract establishes ineligibility | Ineligible (fewer than 5 MVD patients) |
| Habeych ME, Crammond DJ, Gardner P, et al (2014) Intraoperative neurophysiological monitoring of microvascular decompression for glossopharyngeal neuralgia. J Clin Neurophysiol. https://doi.org/10.1097/WNP.0000000000000070 | English | Neurophysiological monitoring in 27 MVD patients (UPMC, January 2008-August 2012) | Full text not obtained; on re-examination the abstract establishes ineligibility | Ineligible (no pain-relief or vessel outcomes reported) |

*Full texts were sought through the institutional library, publishers’ websites and open-access sources, and the search was repeated on journal and publisher platforms (the Chinese Medical Association platform, Chinese open-access journal platforms, J-STAGE and Media Sphera) for every potentially relevant report not retrieved. Every abstract was read in full. Ahn 2024 and Shankar 2020 are meeting abstracts whose supplements were not obtained; the supplement containing the meeting abstract of Segura-Zenón 2022 was obtained, and that report was assessed and excluded at full text (Supplementary Table S2). Nine of the 18 reports are in languages other than English (Chinese 6, French 1, Russian 1, Spanish 1). AICA = anterior inferior cerebellar artery; MVD = microvascular decompression; PICA = posterior inferior cerebellar artery.*

**Supplementary Table S7. Derivation of study-level age, symptom duration and follow-up.**

| **Study** | **Age (years)** | **n** | **Source** | **Duration (years)** | **n** | **Source** | **Follow-up (months)** | **n** | **Source** |
| --- | --- | --- | --- | --- | --- | --- | --- | --- | --- |
| Kondo 1998 | 59.3 | 16 | Reported mean | 5.1 | 16 | Reported mean | 139.2 | 16 | Reported mean |
| Patel 2002 | 50.2 | 217 | Reported mean | 5.7 | 217 | Reported mean | 48.8 | 50 | Mean (50 patients surveyed) |
| Sampson 2004 | 56.4 | 47 | Reported mean | 7.1 | 47 | Reported mean | 155.8 | 29 | Estimated mean from median 152 (range 124-211), 29 patients with long-term follow-up |
| Ferroli 2009 | 55.8 | 31 | Mean from individual patient data | 4.6 | 31 | Reported mean | 90.0 | 31 | Reported mean |
| Kandan 2010 | 52.5 | 21 | Mean (whole 21-patient cohort) | 8.1 | 21 | Mean (whole cohort) | 48.0 | 21 | Mean (whole cohort) |
| Kawashima 2010 | 59.2 | 14 | Reported mean | Not reported |  |  | 78.0 | 14 | Reported mean |
| Gaul 2011 | 54.5 | 19 | Reported mean | 6.5 | 18 | Reported mean | Not reported |  |  |
| Xiong 2012 | 50.4 | 21 | Reported mean | 8.5 | 21 | Reported mean | 40.8 | 21 | Mean (3.4 years) |
| Kim 2017 | 52.4 | 30 | Reported mean | 3.3 | 30 | Mean (39.7 months) | 68.5 | 30 | Reported mean |
| Zhao 2017 | 56.8 | 35 | Mean from individual patient data | 6.3 | 35 | Mean from individual patient data | 132.0 | 30 | Mean (30 patients followed) |
| Wang J 2018 | Not estimable |  | Range only (40-81) | Not reported |  |  | 45.5 | 22 | Estimated mean from median 48 (range 6-72) |
| Xia 2018 | 63.2 | 228 | Reported mean | 4.6 | 228 | Mean (54.7 months) | 54.3 | 228 | Reported mean |
| Zheng X 2020 | 61.6 | 46 | Reported mean | 3.6 | 46 | Reported mean | 38.0 | 46 | Reported mean |
| Du 2021 | 55.3 | 63 | Mean (MVD group) | 4.6 | 63 | Mean (MVD group) | 46.9 | 67 | Reported mean |
| Zheng W 2022 | 59.8 | 97 | Reported mean | 4.5 | 97 | Mean (54.1 months) | Not reported |  |  |
| Wang L 2023 | 62.9 | 39 | Mean from individual patient data (MVD arm) | 3.1 | 39 | Mean from individual patient data (MVD arm) | Not estimable |  | Range only (24-108) |
| Revuelta-Gutierrez 2023 | 58.9 | 20 | Reported mean | 7.6 | 20 | Mean age at surgery minus mean age at onset | 120.4 | 20 | Reported mean |
| Hurth 2024 | 55.0 | 9 | Reported mean | 4.8 | 9 | Reported mean | 15.7 | 9 | Reported mean |
| Joncas 2025 | 61.4 | 29 | Estimated mean from median 65.0 (IQR 52.0-68.0) | 3.5 | 29 | Estimated mean from median 35 months (IQR 9-79) | 65.3 | 29 | Reported mean |
| **Weighted mean (studies; patients)** | **57.3** | **18; 982** |  | **5.1** | **17; 967** |  | **65.9** | **16; 663** |  |

*Means were weighted by the number of patients described. Medians were converted to estimated means with the method of Luo et al. (Stat Methods Med Res 2018;27:1785-1805): for a median with range, w = 4/(4 + n^0.75) and mean ≈ w(minimum + maximum)/2 + (1 − w) × median; for a median with interquartile range, w = 0.7 + 0.39/n and mean ≈ w(Q1 + Q3)/2 + (1 − w) × median. Patient-level means were recomputed from the published patient tables of Ferroli 2009, Zhao 2017 and Wang L 2023 (MVD arm). Studies reporting only a range or no value were not included in the weighted mean.*

**Supplementary Figure S1. Perioperative mortality: forest and funnel plots.**

*(A) Forest plot and (B) funnel plot from MetaAnalysisOnline.com (19 studies, 1,000 patients). Because only 3 of 19 studies recorded a death, the back-transformed pooled estimate is degenerate at this event rate (shown as 0.00), so mortality is reported as the crude rate (5 deaths among 1,000 patients, 0.5%).*

**Supplementary Figure S2. Forest plots of offending-vessel involvement.**

*(A) PICA involvement; (B) vertebral artery involvement; (C) AICA involvement; (D) venous involvement; (E) multiple-vessel conflict. Forest plots from MetaAnalysisOnline.com: squares are study proportions sized by weight with 95% confidence intervals, diamonds are pooled random-effects estimates and red bars 95% prediction intervals. Fifteen studies, 674 patients. Vessels are counted as involvement alone or in combination, so the panels are not mutually exclusive. AICA = anterior inferior cerebellar artery; PICA = posterior inferior cerebellar artery.*

**Supplementary Figure S3. Funnel plots for the eight pooled outcomes.**

**A	Immediate pain relief**

**B	Pain relief at last follow-up**

**C	Recurrence**

**D	PICA involvement**

**E	Vertebral artery involvement**

**F	AICA involvement**

**G	Venous involvement**

**H	Multiple-vessel conflict**

*Funnel plots from MetaAnalysisOnline.com: the Freeman–Tukey double arcsine transformed proportion of each study is plotted against its standard error; the vertical dotted line marks the pooled estimate and the diagonal dotted lines the pseudo 95% confidence limits. Because the efficacy proportions lie close to 1 and the recurrence, AICA and venous proportions close to 0, some asymmetry is expected on statistical grounds alone and should not be interpreted as evidence of publication bias.*
